# Personality changes following first-time psychedelic use in college students in Germany

**DOI:** 10.1038/s44184-026-00228-z

**Published:** 2026-07-10

**Authors:** Constantin Volkmann, Michael Seitz, Ricarda Evens, Moritz Bruno Petzold, Michael Koslowski, Felix Betzler

**Affiliations:** 1 Department of Psychiatry, Charité-Universitätsmedizin Berlin, corporate member of Freie Universität Berlin and Humboldt-Universität zu Berlin, Berlin, Germany; 2 Department of Psychology, Humboldt-Universität zu Berlin, Berlin, Germany; 3 Department Psychologie, MSB Medical School Berlin—Hochschule für Gesundheit und Medizin, Berlin, Germany

**Keywords:** Neuroscience, Psychology, Psychology

## Abstract

Psychedelics may influence personality traits, but longitudinal evidence on first-time use outside clinical settings remains limited. We followed 102 first-time psychedelic users and 1066 never-users over one year among Berlin university students. Personality was assessed with the Big Five Inventory at baseline and follow-up. Linear mixed-effects models showed small relative increases in Openness and decreases in Conscientiousness among first-time users. After adjustment for age, sex, income, psychiatric diagnosis, and baseline substance-use burden, estimates were attenuated but directionally similar (Openness: beta = 0.19, SE = 0.10, *p* = 0.06; Conscientiousness: beta = –0.20, SE = 0.10, *p* = 0.05; FDR-adjusted *p* = 0.16 for both). Change was not clearly different from first-time users of other illicit substances. In exploratory moderation analyses, first-time users with psychiatric diagnoses showed larger Neuroticism reductions. Findings suggest small personality changes after first-time psychedelic use, with limited causal interpretability.

## Introduction

Psychedelic research has experienced a notable renaissance in recent years, driven by evidence suggesting therapeutic benefits in conditions such as depression, anxiety, and substance use disorders^1–3^. Serotonergic psychedelics—most notably psilocybin, lysergic acid diethylamide (LSD), N,N-dimethyltryptamine (DMT), and 5-methoxy-N,N-dimethyltryptamine (5-MeO-DMT)—appear to exert effects on cognition, emotion, and potentially on personality traits^4^. The possibility that psychedelics may alter personality traits has garnered increasing attention, given that personality is traditionally conceptualized as relatively stable yet implicated in mental health and resilience^5,6^.

Personality is commonly conceptualized through the Five-Factor Model (FFM), which organizes traits into Openness, Conscientiousness, Extraversion, Agreeableness, and Neuroticism (OCEAN)^7^. Although personality traits are often considered stable over time, emerging data suggest that personality can shift in response to major life events, including psychedelic experiences^8,9^. These traits are clinically relevant, particularly in relation to resilience and long-term mental health outcomes. For instance, higher Neuroticism is associated with elevated vulnerability to mood and anxiety disorders^10^, whereas increases in Conscientiousness and Agreeableness may strengthen coping strategies and social support^11^. Given these clinical implications, understanding whether such changes occur outside therapeutic settings is important.

Randomized-controlled trials and meta-analyses have linked psychedelic experiences to increases in Openness, with varying effect sizes (Cohen’s d = 0.25–0.85)^4,12–16^. Some non-randomized studies have reported decreases in Neuroticism and increases in Extraversion^17–19^, but findings are heterogeneous. Most studies are limited by small sample sizes, tightly controlled environments (e.g., clinical trials or retreats), or short follow-up periods.

Prior naturalistic and observational studies have reported personality differences or changes following psychedelic use outside clinical settings, including elevated Openness among recreational users, personality changes after ceremonial ayahuasca use, decreases in anxiety and Neuroticism among psilocybin-retreat attendees, and changes in social connectedness and related personality domains in online naturalistic samples^20–25^. Nevertheless, important gaps remain. Few studies have examined first-time psychedelic use longitudinally in young adults, although recent adolescent and emerging-adult studies highlight the developmental relevance of psychedelic use in this age range^26–28^. Moreover, most naturalistic cohorts do not include a non-exposed comparison group, with few exceptions in ritual ayahuasca research^29^. Additionally, evidence is concentrated in a small number of cultural contexts, leaving open the question of cross-cultural reproducibility.

The present study addresses these gaps by following a large cohort of students enrolled at universities across Berlin, Germany, comparing first-time psychedelic users to non-users over one year. This is relevant for public-health discourse because naturalistic psychedelic use has increased in representative US data, including psilocybin and LSD use^30–32^. By assessing participants at baseline and one year later, we investigated whether previously reported increases in Openness or decreases in Neuroticism could be observed in a prospective naturalistic setting. The study, therefore, adds real-world longitudinal evidence from a German student cohort to the literature on psychedelic use and personality change.

## Methods

### Study design and recruitment

This study draws on data from the “Student Drug Survey,” a major observational cohort study examining substance use patterns among university students in Berlin^33,34^. The study was conducted in accordance with the Declaration of Helsinki and received ethics approval from the Charité Universitätsmedizin Berlin (EA1/258/16).

A total of 35 universities in Berlin were contacted, of which 17 (48.6%) agreed to participate by distributing an online questionnaire to their students. An invitation to participate was sent by email to all students of the respective universities. Data collection was carried out between November 3, 2016, and September 30, 2017 (with an additional follow-up period). Informed consent was obtained from all participants before they began the questionnaire. They were informed of their right to withdraw at any time without penalty, and confidentiality and anonymity were ensured throughout the study. The online questionnaire was generated using SoSciSurvey software^35^. The language of the questionnaire was German.

After completing the baseline survey, participants were re-contacted 12 months later to complete a follow-up survey. Both surveys assessed substance use history, demographics, and personality traits via a standardized questionnaire (described below). As an incentive, participants were offered a chance to enter a raffle for one 100 Euro cash prize and five 25 Euro Amazon vouchers.

### Participants

Undergraduate and graduate students enrolled at participating universities were included; on average, respondents were in the fourth semester of their degree. While the vast majority of participants held German nationality, a small minority (*n* = 38; 0.4%) reported multiple nationalities. No specific exclusion criteria were applied, aside from requiring participants to provide informed consent and be at least 18 years old.

### Assessments

Personality traits were measured using the Short 15-item Big Five Inventory (BFI-S)^36^. The BFI-S assesses Openness to Experience, Conscientiousness, Extraversion, Agreeableness, and Neuroticism via 15 items, each rated on a 7-point scale. For each trait, scores were calculated at baseline and at follow-up, allowing for within-person comparisons over time.

For each of 20 different substances (see Supplementary Table 1), participants selected one mutually exclusive response option describing their use history: “Never used”, “Used once”, “Used in the past 12 months”, or “Used in the past month”. We classified LSD, psilocybin, and DMT as psychedelics, and all other illicit drugs as non-psychedelics.

Demographic variables (age, sex, and education level), income, and psychiatric diagnoses were self-reported. Sex was assessed with three options (*male*, *female*, *other*). Due to the small number of participants selecting “other”, these cases were treated as missing in analyses where sex was included as a moderator. Psychiatric history was assessed by asking participants whether they had ever received a psychiatric diagnosis (yes/no), with the option to specify the diagnosis. Ethnicity was not assessed.

### Data analysis

All statistical analyses were performed in R Studio (Version 2022.12.0 + 353). Respondents were excluded if they had more than 20% missing answers, incomplete questionnaires, or implausibly short response times^33^. Personality scores were z-standardized to improve comparability across dimensions. Participants were categorized based on their psychedelic use between baseline and follow-up.

1. **Never Users:** Participants who indicated having “Never used” any psychedelic at baseline and at follow-up regardless of other substance use.
2. **First-Time Psychedelic Users**: Participants who indicated having “Never used” any psychedelic at baseline and indicated “Used once”, “Used in the past 12 months”, or “Used in the past month” for at least one psychedelic substance at follow-up, regardless of other substance use.
  A third comparison group was created for exploratory comparison analyses:
3. **Other Drug Users**: Participants who indicated having “Never used” any psychedelic at baseline and at follow-up and reported “Used in the past 12 months” or “used in the past month” for at least one non-psychedelic drug at follow-up for which they had indicated “Never used” at baseline.

Baseline demographic characteristics between groups were compared using independent samples t-tests for continuous variables and Fisher’s exact tests for categorical variables. False discovery rate (FDR) correction was applied to account for multiple comparisons.

To assess personality trait change over time, repeated-measures linear mixed-effects models were fit using the lme4 package^37^, with random intercepts for participant ID to account for within-subject correlation. Separate models were estimated for each trait. For the primary analyses, two models were fitted per trait: an unadjusted model including group (first-time psychedelic user vs. never user), time (baseline vs. follow-up), and their interaction, and an adjusted model additionally including age, sex, income, baseline psychiatric diagnosis, and the standardized number of different substances used at baseline as covariates. Sex was coded from the original three-category sex item; participants selecting other were treated as missing in models including binary sex because this subgroup was very small. This resulted in a total of 10 models in the primary analysis.

The group × time interaction represents the primary parameter of interest and quantifies differential change in personality traits from baseline to follow-up between groups. Baseline and follow-up personality scores were modeled jointly as repeated outcome measures, and baseline trait levels were therefore not included as covariates in these models.

*P*-values for fixed effects were obtained using Satterthwaite’s approximation for degrees of freedom as implemented in the lmerTest package^38^. False discovery rate (FDR) correction was applied across the five primary models (one per Big Five trait) using the Benjamini-Hochberg method. Unadjusted and adjusted models were corrected separately, each involving 5 models. The same FDR correction approach was applied to the comparison analyses involving first-time users of other illicit substances and the moderation analyses.

We report effect estimates with 95% confidence intervals and exact *p*-values to quantify the magnitude and uncertainty of associations. We avoid dichotomizing findings solely as statistically significant or non-significant. False discovery rate-adjusted *p*-values are provided to aid interpretation in the context of multiple testing.

Moderation analyses were conducted within the same linear mixed-effects modeling framework as the primary analyses. Separate models were estimated for each of the five Big Five traits, including three-way interaction terms (group × time × moderator). For each moderator, two models per trait were fitted (unadjusted and adjusted), resulting in a total of 10 models per moderator. Adjusted models included the same covariates as the primary analyses, excluding the moderator itself when appropriate to avoid redundancy. In additional exploratory models, we compared psychedelic users to first-time users of non-psychedelic substances to assess the specificity of effects.

To address missing data in predictor variables, multiple imputation by chained equations (MICE) was used. Specifically, predictive mean matching was employed via the mice package in R to generate five imputed datasets (m = 5). Rubin’s Rules were then used to pool the model estimates, standard errors, and confidence intervals across the five imputations, allowing for unbiased inference that reflects the uncertainty due to missing data.

The baseline substance-count covariate was used instead of a binary baseline drug-use indicator because substance-use burden was a bounded, non-normally distributed count variable and differed between groups at baseline.

Personality scores were z-standardized prior to analysis. As a result, regression coefficients can be interpreted as standardized effect sizes, representing the difference in standard deviation units between groups. Following conventional benchmarks, values of approximately 0.2, 0.5, and 0.8 may be interpreted as small, medium, and large effects, respectively^39^.

## Results

Out of a total of 9351 participants that completed the baseline survey, 1443 also completed the follow-up assessment and were eligible for longitudinal analyses. Within this subsample, 1066 participants reported no psychedelic use (“never users”), 102 reported first-time use between assessments (“first-time users”), and 229 reported prior psychedelic use at baseline. An additional 46 participants with inconsistent reporting of psychedelic use across assessments were excluded from analysis. The present analyses focus on comparisons between first-time users and never users. The baseline characteristics of these two groups are presented in Table 1. While the groups were largely comparable across most variables, a higher proportion of males was observed among first-time users compared to never users (59.8% vs. 43.9%, FDR *p* = 0.008). First-time users also reported higher income at the second assessment, although this difference was less clearly distinguishable after false discovery rate (FDR) adjustment. Among participants reporting a psychiatric diagnosis at baseline, the most common specific coded diagnostic categories were depressive episodes (*n* = 68), other anxiety disorders (*n* = 14), hyperkinetic disorders (*n* = 12), eating disorders (*n* = 9), and reactions to severe stress or adjustment disorders (*n* = 8). An additional 36 participants were coded under a non-specific multiple/comorbid-diagnosis category; because this code does not identify a distinct diagnosis, it is reported separately. The full coded diagnosis breakdown is provided in Supplementary Table 4.

**Table 1:** Characteristics of first-time psychedelic users (*n* = 102) and never users (*n* = 1066)

Variable	First-time users (*n* = 102)	Never users (*n* = 1066)	Raw *p* value	FDR *p* value
**Age**	23.52 (4.15)	23.35 (4.43)	0.693	0.693
**Male (%)**	59.80%	43.90%	0.001	0.008
**PsychDx1 (%)**	19.61%	15.76%	0.323	0.517
**PsychDx2 (%)**	19.61%	17.45%	0.587	0.679
**Income1 (€/month)**	853 (474)	826 (620)	0.594	0.679
**Income2 (€/month)**	1110 (746)	936 (519)	0.024	0.094
**No. of substances used at baseline (median [Q1, Q3])**	3^2,4^	2^1,2^	<0.001	<0.001
**No. of substances used at follow-up (median [Q1, Q3])**	6 [4, 8.75]	2^1,2^	<0.001	<0.001

Values are mean (SD), percentages, or median [Q1, Q3], as appropriate. Raw *p* values were calculated using independent-samples *t* tests for approximately normally distributed continuous variables, Wilcoxon rank-sum tests for substance-count variables, and Fisher’s exact tests for categorical variables. Adjusted *p* values were derived using the Benjamini-Hochberg procedure to control the false discovery rate (FDR). Follow-up substance counts are reported descriptively to characterize group differences over time. PsychDx1/2: Psychiatric diagnosis at baseline/follow-up. Analogous nomenclature for income and substance use.

### Personality change

**Fig. 1:**
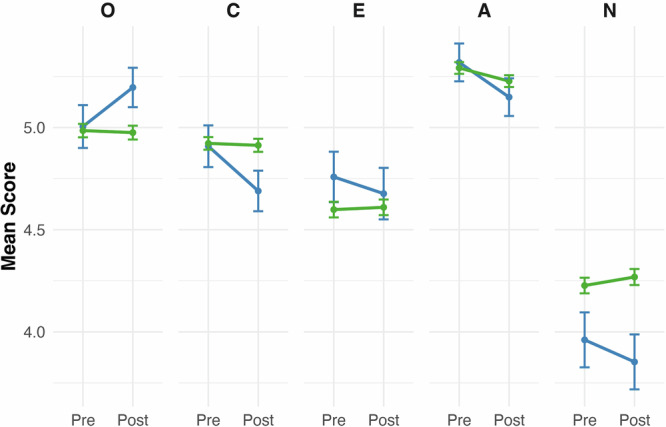
First-time psychedelic users and never-users show different longitudinal patterns across Big Five trait scores. Points represent raw mean BFI-S trait scores at baseline (Pre) and follow-up (Post), and vertical error bars represent standard errors. Panels show Openness (O), Conscientiousness (C), Extraversion (E), Agreeableness (A), and Neuroticism (N), respectively. Blue circles and lines represent first-time psychedelic users; green circles and lines represent never-users. Regression analyses were conducted on z-standardized scores; plotted values are raw scores for descriptive visualization.

Mean personality trait scores at baseline and follow-up for first-time psychedelic users and never users are visualized in Fig. 1. Unadjusted mixed-effects models showed a small increase in Openness (estimate = 0.201, *p* = 0.04) and a small decrease in Conscientiousness (estimate = -0.210, *p* = 0.03). The observed group differences for Extraversion, Agreeableness, and Neuroticism were smaller and less precise, with confidence intervals including 0. In adjusted models including age, sex, income, baseline psychiatric diagnosis, and baseline number of substances used, estimates for Openness and Conscientiousness remained similar in direction but were attenuated (Openness: estimate = 0.188, *p* = 0.06; Conscientiousness: estimate = –0.201, *p* = 0.05; FDR-adjusted *p* = 0.16 for both). The results of the unadjusted and adjusted mixed-effects models are summarized in Table 2.

**Table 2:** Results from ten linear mixed-effects models comparing first-time psychedelic users with never users (two models per personality trait: one unadjusted and one adjusted for covariates)

	Unadjusted	*p* value (FDR)	Adjusted	*p* value (FDR)
**Openness**	0.20 [0.01, 0.39]	0.04 (0.11)	0.19 [–0.01, 0.38]	0.06 (0.16)
**Conscientiousness**	–0.21 [–0.40, –0.02]	0.04 (0.11)	–0.20 [–0.40, –0.004]	0.05 (0.16)
**Neuroticism**	–0.15 [–0.38, 0.08]	0.20 (0.32)	–0.13 [–0.36, 0.11]	0.29 (0.36)
**Agreeableness**	–0.11 [–0.29, 0.08]	0.26 (0.32)	–0.12 [–0.30, 0.07]	0.21 (0.34)
**Extraversion**	–0.09 [–0.30, 0.12]	0.39 (0.39)	–0.03 [–0.25, 0.18]	0.76 (0.76)

For each Big Five trait, models included fixed effects for group (first-time user vs. never user), time (baseline vs. follow-up), and the group x time interaction, with random intercepts for participant ID. Adjusted models additionally included age, sex, baseline psychiatric diagnosis, baseline income, and the standardized number of different substances used at baseline as covariates. Estimates represent the group x time interaction and therefore reflect differential change over time between groups. *P* values were obtained using Satterthwaite’s approximation for degrees of freedom and were corrected across the five trait-specific interaction terms using the false discovery rate (FDR).

Missing data were limited to baseline income (*n* = 27) and sex (*n* = 9). In our main analysis, we used listwise deletion for participants with missing covariate data. As a robustness check, we conducted sensitivity analyses using multiple imputation via chained equations (MICE) with five imputed datasets, which yielded nearly identical effect estimates (see Supplementary Table 3).

The adjusted results therefore support the same qualitative pattern as the unadjusted models, but with greater uncertainty after accounting for sex and baseline substance-use burden.

Taken together, the primary models suggest small differences in Openness and Conscientiousness after first-time psychedelic use, but the magnitude and robustness of these associations should be interpreted cautiously.

### Comparison with other illicit drug initiators

**Fig. 2:**
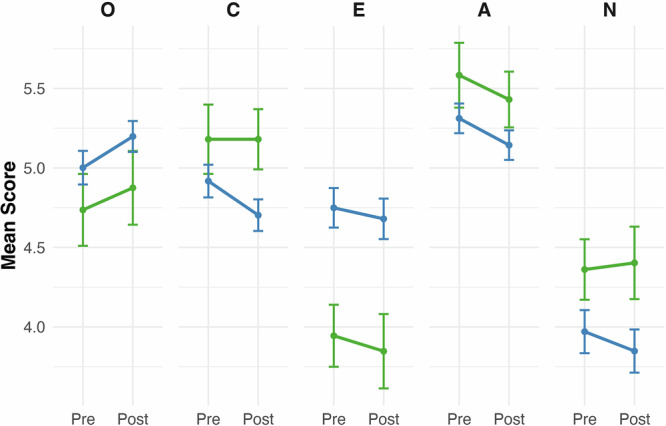
First-time psychedelic users and first-time users of other illicit drugs show descriptive Big Five trait trajectories over one year. Points represent raw mean BFI-S trait scores at baseline (Pre) and follow-up (Post), and vertical error bars represent standard errors. Panels a-e show Openness (O), Conscientiousness (C), Extraversion (E), Agreeableness (A), and Neuroticism (N), respectively. Blue circles and lines represent first-time psychedelic users; green circles and lines represent first-time users of other illicit drugs. Regression analyses were conducted on z-standardized scores; plotted values are raw scores for descriptive visualization.

To discern whether the observed effects were specific to psychedelics or merely reflected general novelty-seeking behavior, we compared first-time psychedelic users to individuals who had tried any other (non-psychedelic) illicit substance for the first time between the assessments. Figure 2 shows mean personality trait scores at baseline and follow-up for first-time psychedelic users compared to first-time users of another illicit substance. The previously observed group differences in Openness and Conscientiousness were attenuated (see Table 3). The comparison group comprised participants who had never used psychedelics at baseline or follow-up and who reported first-time use of at least one non-psychedelic illicit substance between assessments; however, this group was small (*n* = 24).

**Table 3:** Results from ten linear mixed-effects models comparing first-time psychedelic users with first-time users of other illicit drugs (two models per personality trait: one unadjusted and one adjusted for covariates)

	Unadjusted	*p* value (FDR)	Adjusted	*p* value (FDR)
**Openness**	0.06 [–0.43, 0.55]	0.82 (0.95)	0.08 [–0.45, 0.61]	0.76 (0.94)
**Conscientiousness**	–0.21 [–0.73, 0.30]	0.42 (0.95)	–0.02 [–0.58, 0.54]	0.94 (0.94)
**Neuroticism**	0.03 [–0.57, 0.62]	0.93 (0.95)	0.14 [–0.51, 0.79]	0.67 (0.94)
**Agreeableness**	–0.02 [–0.49, 0.46]	0.95 (0.95)	–0.12 [–0.63, 0.39]	0.65 (0.94)
**Extraversion**	–0.16 [–0.81, 0.48]	0.62 (0.95)	–0.20 [–0.90, 0.50]	0.58 (0.94)

For each Big Five trait, models included fixed effects for group (first-time psychedelic use vs. first-time other illicit drug use), time (baseline vs. follow-up), and the group x time interaction, with random intercepts for participant ID. Adjusted models additionally included age, sex, baseline psychiatric diagnosis, baseline income, and baseline non-psychedelic drug use as covariates. Estimates represent the group x time interaction and therefore reflect differential change over time between groups. *P* values were obtained using Satterthwaite’s approximation for degrees of freedom and were corrected across the five trait-specific interaction terms using the false discovery rate (FDR).

### Subgroup analyses

To explore whether demographic or clinical factors modified the observed associations, we conducted a series of post hoc analyses that included sex and psychiatric diagnoses as potential effect modifiers. Supplementary Table 2 shows the complete moderation results, and Supplementary Fig. 1 provides additional descriptive trajectories by psychedelic-use group and baseline psychiatric diagnosis.

### Sex differences

In models including a three-way interaction between group (first-time user vs. never-user), sex, and time, sex did not clearly moderate personality change. Although point estimates for Openness differed by sex, confidence intervals included zero, and the estimates were imprecise. Using the same first-time-user minus never-user contrast as in the primary models, the interaction estimate for Openness was 0.35 (SE = 0.20, FDR-adjusted *p* = 0.29) in the unadjusted model and 0.39 (SE = 0.21, FDR-adjusted *p* = 0.28) in the adjusted model. Interaction estimates for the remaining traits were small and imprecise. Overall, these exploratory analyses did not provide clear evidence that sex moderated personality change trajectories.

### Moderation by psychiatric diagnosis

**Fig. 3:**
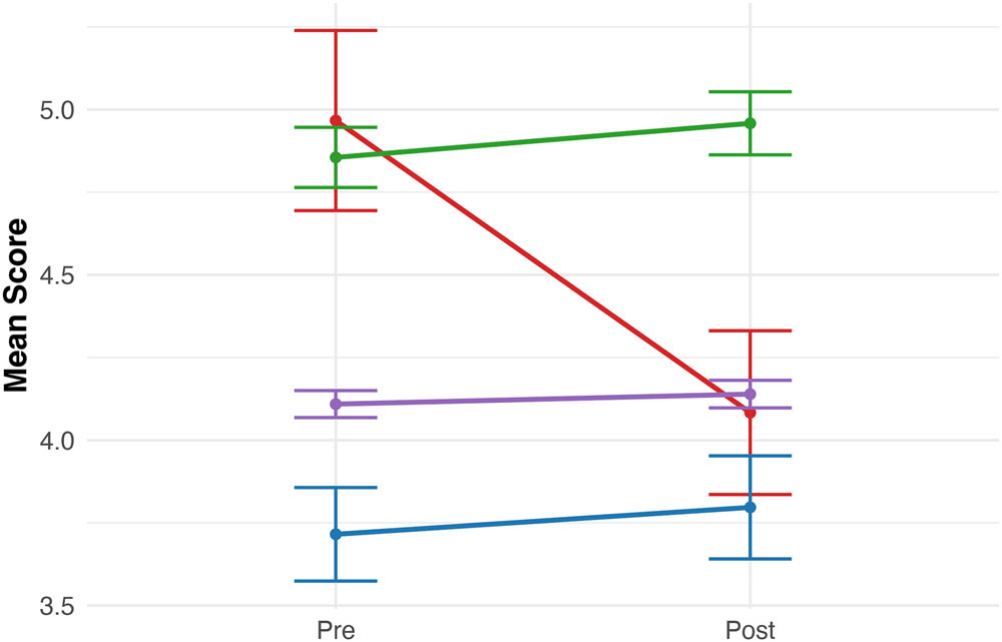
Baseline psychiatric diagnosis is associated with different Neuroticism trajectories by psychedelic-use group. Points represent raw mean BFI-S Neuroticism scores at baseline (Pre) and follow-up (Post), and vertical error bars represent standard errors. Red represents first-time psychedelic users with a baseline psychiatric diagnosis; green represents never-users with a baseline psychiatric diagnosis; blue represents first-time psychedelic users without a baseline psychiatric diagnosis; and purple represents never-users without a baseline psychiatric diagnosis. Regression analyses were conducted on z-standardized scores; plotted values are raw scores for descriptive visualization. Baseline psychiatric diagnosis is associated with different Neuroticism trajectories by psychedelic-use group.

We next investigated whether a history of psychiatric diagnosis moderated personality change by including a three-way interaction between group, psychiatric diagnosis at baseline, and time. For Neuroticism, there was evidence that baseline psychiatric diagnosis moderated change among first-time psychedelic users (Fig. 3). Using the same first-time-user minus never-user contrast as in the primary models, the adjusted model yielded an interaction estimate of –1.09 (SE = 0.30, FDR-adjusted *p* = 0.001), and the unadjusted model showed a similar estimate of –1.04 (SE = 0.29, FDR-adjusted *p* = 0.002). This coefficient represents an additional decrease in Neuroticism for diagnosed first-time users relative to all other combinations of group and diagnosis. Because personality outcomes were z-standardized, this coefficient corresponds to approximately one standard deviation unit in standardized Neuroticism change rather than one raw scale point.

For Openness, the adjusted estimate was –0.49 (SE = 0.25, FDR-adjusted *p* = 0.13), with uncertainty intervals compatible with smaller or negligible effects. For Conscientiousness, Extraversion, and Agreeableness, interaction estimates were small and inconsistent, with FDR-adjusted *p* > 0.6 in both models (see Supplementary Fig. 1).

## Discussion

In this longitudinal, naturalistic study of German university students, we investigated whether first-time psychedelic use was associated with changes in Big Five personality traits. Our primary analyses suggest a small increase in Openness and a small decrease in Conscientiousness over a one-year period in those who used psychedelics for the first time, compared to non-users. These estimates remained directionally similar but were attenuated after adjustment for demographic, clinical, and baseline substance-use covariates. Other traits-Neuroticism, Extraversion, and Agreeableness-did not show meaningful or consistent change in the primary models.

These findings align with prior studies linking psychedelic experiences to increases in Openness, a trait associated with curiosity, imagination, and receptivity to new experiences^12,20,40,41^. The present findings also sit within a broader naturalistic literature reporting personality-related differences or changes after recreational psychedelic use, ceremonial ayahuasca use, psilocybin retreat attendance, and other non-clinical psychedelic experiences^20–25^. Our study adds to this work in two specific ways: it focuses on first-time psychedelic use and it includes a large non-exposed comparison group drawn from the same student cohort. At the same time, the observed primary estimates were small and became more uncertain after adjustment for sex, psychiatric diagnosis, income, and baseline substance-use burden. This pattern suggests that psychedelic use may be one marker of broader behavioral or psychosocial differences among students rather than an isolated cause of personality change. Because we did not assess motives, dose, timing, setting, or subjective intensity of psychedelic use, we cannot determine whether experiences were recreational, self-exploratory, therapeutic, or otherwise motivated; prior survey work suggests that naturalistic motives may include mental-health and well-being aims as well as other reasons^42^.

The decrease in Conscientiousness should be interpreted cautiously. It was small in standardized magnitude and contrasts with some clinical studies that report stability or increases in Conscientiousness after structured psychedelic interventions^20,41^. This difference may reflect sample, measurement, or contextual factors.

To assess the specificity of these changes, we compared the psychedelic group to individuals who tried a different illicit substance (non-psychedelic) for the first time during the same time window. In this comparison, the changes in Openness and Conscientiousness were markedly attenuated. Because baseline and follow-up personality scores were modeled jointly within a longitudinal framework, the observed group × time interactions cannot be attributed solely to static pre-existing differences in Openness. Instead, the findings suggest that the broader context of engaging in novel behaviors may partly account for the observed effects. This interpretation aligns with the known overlap between Openness and constructs like sensation-seeking, and it supports a more cautious interpretation of psychedelic-specific mechanisms.

We further explored whether baseline sex and psychiatric diagnosis moderated personality change trajectories. These analyses were exploratory and are interpreted primarily in terms of effect size and uncertainty.

Sex did not clearly moderate personality change trajectories. Although point estimates for Openness differed by sex, confidence intervals included zero; therefore, these analyses should not be taken as evidence of sex-specific effects.

The most pronounced exploratory moderation signal was observed among participants with a baseline psychiatric diagnosis, for whom first-time psychedelic use was associated with a larger reduction in standardized Neuroticism than among participants without such a diagnosis. Neuroticism, which is associated with emotional instability and vulnerability to stress, is clinically relevant in the context of psychedelic therapy. Because these findings are observational and exploratory, they should be interpreted as hypothesis-generating rather than causal evidence.

This finding resonates with clinical literature reporting reductions in Neuroticism among patients treated with psychedelics for depression or anxiety^19^ and with naturalistic evidence suggesting personality-related changes after ceremonial, retreat-based, or other non-clinical psychedelic use^20–25^. Potential mechanisms such as emotional breakthroughs and ego dissolution have been proposed in therapeutic contexts^43^, but they were not measured in the present study.

Several limitations should be acknowledged. First, psychedelic use was not randomized and self-reported, introducing potential biases related to motivation, memory, dosage, or context. The exact timing of first psychedelic use within the one-year follow-up interval was not assessed, so we cannot determine whether observed personality differences reflect enduring change or more recent/subacute effects. We also collapsed across different psychedelic substances, which may differ in pharmacology, subjective effects, dose, and cultural context of use. Second, our binary measure of psychiatric diagnosis likely underestimated the heterogeneity of clinical presentations. Third, while we adjusted for several demographic and clinical covariates and modeled personality longitudinally, residual confounding by unmeasured factors (e.g., major life events, context, and intention of use) remains possible. Ethnicity was not assessed in the present study, limiting our ability to examine potential cultural or ethnic differences in psychedelic use and associated personality change. The direction of causality is also uncertain: personality changes due to other factors may have increased the likelihood of psychedelic use rather than resulting from it. Fourth, recruitment via an online survey may have introduced selection bias if individuals with particular traits, stronger interest in substance use, or more salient psychedelic experiences were more likely to participate. Attrition may also have biased longitudinal estimates if follow-up completion differed by personality, psychiatric symptoms, or substance-use patterns, as attrition in longitudinal studies can be related to personality and other participant characteristics^44,45^; we therefore report baseline-to-follow-up attrition in the Results. Because the analytic dataset contained participants with linked baseline and follow-up personality data, we could not fully model predictors of dropout among baseline-only respondents in the revised analyses. Finally, the one-year follow-up period is relatively short in the context of personality development.

Despite these caveats, our study adds to a growing body of evidence suggesting that psychedelics may be linked to personality changes in naturalistic settings. Importantly, it underscores the value of longitudinal, real-world data to complement clinical trials. Future work should include repeated follow-ups, incorporate measures of experience quality and intensity, and better differentiate between trait change and temporary state shifts.

Our findings provide preliminary support that first-time psychedelic use may be associated with small personality changes, particularly in Openness and Conscientiousness, although these estimates were attenuated after adjustment for sex and baseline substance-use burden. Exploratory moderation analyses suggested that psychiatric history may be relevant for Neuroticism change, whereas sex did not clearly moderate personality trajectories. Because psychedelic use was observational, self-reported, and not temporally localized within the follow-up interval, causal interpretation remains limited.

## Supplementary information

Supplementary information

## Data Availability

The data supporting the findings of this study are available from the corresponding author upon reasonable request. Data are not publicly available because participants did not consent to unrestricted public data sharing and the dataset contains sensitive information on mental health and substance use. The R analysis code used for this study is available from the corresponding author upon reasonable request.

## Code availability

The R analysis code used for this study is available from the corresponding author upon reasonable request.
