# Supplementary material for: Personality changes following first-time psychedelic use in college students in Germany
Source: Npj Ment Health Res. 2026 Jul 10;5:34. doi: 10.1038/s44184-026-00228-z (PMC13350684; doi:10.1038/s44184-026-00228-z)
Supplement: Supplementary file 1 — Supplementary information [file 44184_2026_228_MOESM1_ESM.pdf]

Supplementary Information

Open minds: Changes in personality traits after first intake of psychedelics in college students.

Assessment prompt	Psychoactive substance	Response options
For each substance, participants indicated whether and when they had used it. Medications taken under medical prescription were not to be reported.	Alcohol	Never used; used once/ever; used in the past 12 months; used in the past month.
	Cannabis (weed, marijuana, hashish)	
	Amphetamine (speed, pep)	
	Methamphetamine (crystal meth)	
	MDMA (ecstasy, pills, crystals or tablets)	
	LSD (acid, tabs)	
	Heroin	
	Opioid-based painkillers (e.g., Tramal, Tilidin)	
	Cocaine	
	Psilocybin (mushrooms, magic mushrooms)	
	Synthetic cannabinoids (Spice or other herbal blends)	
	Synthetic cathinones (bath salts or similar)	
	Methylphenidate (e.g., Ritalin, Medikinet)	
	Modafinil (e.g., Vigil)	
	Benzodiazepines (e.g., Valium, Tavor)	

	Amyl nitrite (poppers)	
	Crack	
	DMT (ayahuasca)	
	GHB/GBL (G, liquid ecstasy)	
	Ketamine (keta, K)	
	Other substances	
	Free-text entry for other substances	

Supplementary Table 1. Substance-use assessment items and response options. The table shows the psychoactive substances included in the baseline substance-use assessment, the instruction given to participants, and the response options used to code lifetime and recent use.

Trait	Moderator	Coefficient (unadjusted)	p value (FDR)	Coefficient (adjusted)	p value (FDR)
Sex moderation models: psychedelic-use group x time x sex (male vs female)					
O	Sex	0.35 [-0.05, 0.76]	.08 (.29)	0.39 [-0.01, 0.80]	.06 (.28)
C	Sex	-0.23 [-0.63, 0.18]	.27 (.45)	-0.23 [-0.64, 0.17]	.26 (.44)
E	Sex	-0.35 [-0.79, 0.09]	.12 (.29)	-0.34 [-0.78, 0.10]	.13 (.33)
A	Sex	-0.13 [-0.51, 0.25]	.50 (.62)	-0.12 [-0.50, 0.26]	.53 (.66)
N	Sex	-0.07 [-0.54, 0.40]	.77 (.77)	-0.06 [-0.54, 0.42]	.80 (.80)
Psychiatric-diagnosis moderation models: psychedelic-use group x time x baseline psychiatric diagnosis (yes vs no)					
O	PsychDx	-0.50 [-0.99, -0.01]	.05 (.11)	-0.49 [-0.98, 0.00]	.05 (.13)
C	PsychDx	0.11 [-0.38, 0.61]	.65 (.65)	0.11 [-0.39, 0.60]	.67 (.67)
E	PsychDx	0.25 [-0.29, 0.78]	.37 (.62)	0.18 [-0.37, 0.72]	.53 (.67)
A	PsychDx	-0.11 [-0.57, 0.35]	.64 (.65)	-0.10 [-0.57, 0.36]	.67 (.67)
N	PsychDx	-1.04 [-1.61, -0.46]	<.001 (.002)	-1.09 [-1.67, -0.51]	<.001 (.001)

Supplementary Table 2. Exploratory moderation analyses by sex and baseline psychiatric diagnosis. Rows report three-way interaction terms from separate linear mixed-effects models for each Big Five trait. Estimates are coded as the first-time psychedelic user versus never-user group-by-time effect, further contrasted by the moderator. Thus, for sex, positive estimates indicate a larger relative first-time-user change in males than females; for psychiatric diagnosis, negative estimates indicate a smaller or more negative relative first-time-user change among participants with versus without a baseline psychiatric diagnosis. Sex moderation comprised 10 models (five unadjusted and five adjusted models); psychiatric-diagnosis moderation comprised 10 models (five unadjusted and five adjusted models). Adjusted models included the same covariate set as the primary analyses, excluding

the moderator itself where appropriate to avoid redundancy. O = Openness; C = Conscientiousness; E = Extraversion; A = Agreeableness; N = Neuroticism; PsychDx = baseline psychiatric diagnosis.

Trait	Coefficient (adjusted)	p value (FDR)
O	0.20 [0.01, 0.39]	.041 (.102)
C	-0.21 [-0.40, -0.02]	.034 (.102)
E	-0.09 [-0.30, 0.12]	.389 (.389)
A	-0.11 [-0.29, 0.08]	.255 (.319)
N	-0.15 [-0.38, 0.08]	.198 (.319)

Supplementary Table 3. Sensitivity analysis after multiple imputation by chained equations (MICE). Missing values for income and sex were imputed before fitting the adjusted linear mixed-effects models for each Big Five trait. Estimates represent the first-time psychedelic user versus never-user group-by-time interaction.

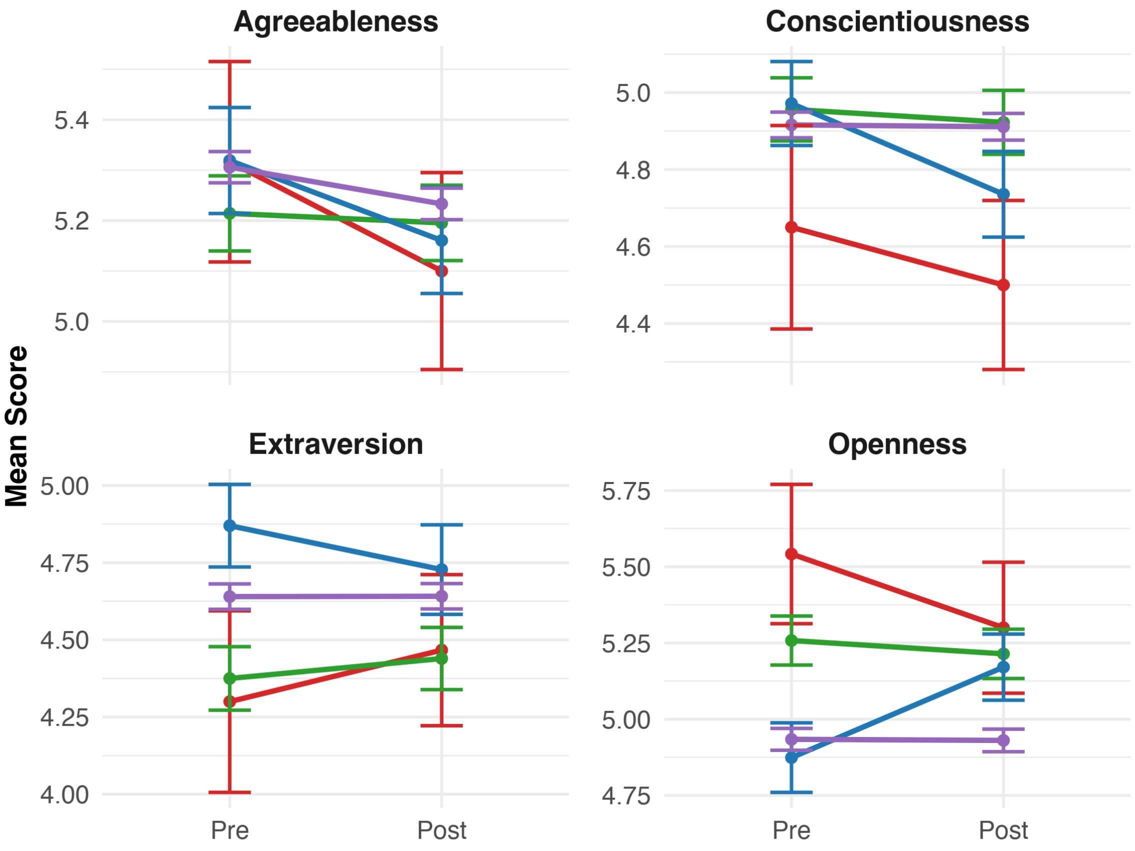

Supplementary Figure 1. Baseline psychiatric diagnosis stratifies descriptive trajectories for Agreeableness, Conscientiousness, Extraversion, and Openness. Points represent raw mean BFI-S trait scores at baseline (Pre) and follow-up (Post), and vertical error bars represent standard errors. Facets show Agreeableness, Conscientiousness, Extraversion, and Openness. Red circles and lines represent first-time psychedelic users with a baseline psychiatric diagnosis; green circles and lines represent never-users with a baseline psychiatric diagnosis; blue circles and lines represent first-time psychedelic users without a baseline psychiatric diagnosis; and purple circles and lines represent never-users without a baseline psychiatric diagnosis. Dx indicates baseline psychiatric diagnosis. Neuroticism is shown in Figure 3 of the main manuscript.

**Coded category**	**English description used for reporting**	**n**
depressive episode	Depressive episodes	68
komorbide störungen	Non-specific multiple/comorbid-diagnosis category	36
blank/missing	Diagnosis endorsed but no codable category provided	21
andere angststörungen	Other anxiety disorders	14
hyperkinetische störungen	Hyperkinetic disorders	12
essstörungen	Eating disorders	9
reaktionen auf schwere belastungen und anpassungsstörungen	Reactions to severe stress or adjustment disorders	8
phobische störungen	Phobic disorders	4
somatoforme störungen	Somatoform disorders	4
anhaltende affektive störung	Persistent affective disorder	2
schizophrenie	Schizophrenia	2
spezifische persönlichkeitsstörungen	Specific personality disorders	2
abnorme gewohnheiten und störungen der impulskontrolle	Abnormal habits and impulse-control disorders	1
alkohol	Alcohol-related disorder	1
andere neurotische störungen	Other neurotic disorders	1
bipolare affektive störung	Bipolar affective disorder	1
multipler gebrauch / andere	Multiple substance use / other	1

zwangsstörung	Obsessive-compulsive disorder	1

Supplementary Table 4. Coded psychiatric-diagnosis categories among participants with a baseline psychiatric diagnosis (PsychDx1 = 1) in the analytic first-time psychedelic user and never-user sample. Categories are based on the coded a304_02 variable. The non-specific multiple/comorbid-diagnosis category and blank/missing entries are reported for transparency but are not interpreted as distinct diagnoses.

STROBE checklist for revised observational cohort report

Manuscript: Personality changes following first-time psychedelic use in college students in Germany

This checklist is provided as a related submission file to support transparent reporting of the revised longitudinal observational cohort analysis.

STROBE item	Where addressed in revised manuscript
1. Title/abstract identifies study design and gives balanced summary	Abstract; Keywords
2-3. Background and objectives	Introduction
4. Study design	Methods: Study Design and Recruitment
5. Setting	Methods: Study Design and Recruitment
6. Participants and eligibility	Methods: Participants; Results
7. Variables	Methods: Assessments; Methods: Data Analysis
8. Data sources/measurement	Methods: Assessments; Supplementary Table 1
9. Bias	Discussion
10. Study size	Results; Table 1
11. Quantitative variables	Methods: Data Analysis
12. Statistical methods	Methods: Data Analysis; Supplementary Table 3
13. Participants/flow	Results
14. Descriptive data	Table 1; Results
15. Outcome data	Results; Tables 2-3; Supplementary Table 2
16. Main results	Results; Tables 2-3; Figure Legends
17. Other analyses	Results: Comparison with Other Illicit Drug Initiators and Subgroup analyses; Supplementary Tables 2-3
18. Key results	Discussion
19. Limitations	Discussion
20. Interpretation	Discussion
21. Generalisability	Discussion

22. Funding	Acknowledgements
